# Integration, abundance, and transmission of mutations and transgenes in a series of CRISPR/Cas9 soybean lines

**DOI:** 10.1186/s12896-020-00604-3

**Published:** 2020-02-24

**Authors:** Jean-Michel Michno, Kamaldeep Virdi, Adrian O. Stec, Junqi Liu, Xiaobo Wang, Yer Xiong, Robert M. Stupar

**Affiliations:** 1 Bioinformatics and Computational Biology Program, University of Minnesota, Minneapolis, MN USA; 2 Department of Agronomy and Plant Genetics, University of Minnesota, 1991 Upper Buford Circle, 411 Borlaug Hall, Saint Paul, MN 55108 USA; 3 School of Agronomy, Anhui Agricultural University, Hefei, China

**Keywords:** Soybean, Transgenic, CRISPR, Transmission, Editing

## Abstract

**Background:**

As with many plant species, current genome editing strategies in soybean are initiated by stably transforming a gene that encodes an engineered nuclease into the genome. Expression of the transgene results in a double-stranded break and repair at the targeted locus, oftentimes resulting in mutation(s) at the intended site. As soybean is a self-pollinating species with 20 chromosome pairs, the transgene(s) in the T0 plant are generally expected to be unlinked to the targeted mutation(s), and the transgene(s)/mutation(s) should independently assort into the T1 generation, resulting in Mendellian combinations of transgene presence/absence and allelic states within the segregating family. This prediction, however, is not always consistent with observed results.

**Results:**

In this study, we investigated inheritance patterns among three different CRISPR/Cas9 transgenes and their respective induced mutations in segregating soybean families. Next-generation resequencing of four T0 plants and four T1 progeny plants, followed by broader assessments of the segregating families, revealed both expected and unexpected patterns of inheritance among the different lineages. These unexpected patterns included: (1) A family in which T0 transgenes and mutations were not transmitted to progeny; (2) A family with four unlinked transgene insertions, including two respectively located at paralogous CRISPR target break sites; (3) A family in which mutations were observed and transmitted, but without evidence of transgene integration nor transmission.

**Conclusions:**

Genome resequencing provides high-resolution of transgene integration structures and gene editing events. Segregation patterns of these events can be complicated by several potential mechanisms. This includes, but is not limited to, plant chimeras, multiple unlinked transgene integrations, editing of intended and paralogous targets, linkage between the transgene integration and target site, and transient expression of the editing reagents without transgene integration into the host genome.

## Background

Modern genome engineering provides the ability to make targeted modifications to genomes. Some of the most popular systems for genome engineering involve delivering a reagent to the cell that induces a double-stranded break (DSB) at a specific DNA sequence, thereby initiating the repair/modification process. Reagent platforms include zinc-finger nucleases and TAL effector nucleases, which can each be engineered as proteins that recognize and create DSBs at specific DNA sequences. These platforms have been used to modify genes in numerous different organisms, including plant species [1–6]. More recently, CRISPR/Cas9 has become a popular genome engineering platform, and has been used across a variety of species due to its ease of construction and range of sequences it is able to target [7–9]. The plant research community has rapidly adopted the CRISPR/Cas9 system, including as a tool for modifying and enhancing different crop species [10–18]. This type of genome editing/engineering provides a toolkit for modifying DNA in a gene-specific manner, allowing researchers, geneticists, and breeders to move beyond the ordinary boundaries of germplasm and genetic variation.

In crop plant species, the majority of trait-driven editing applications have focused on creating targeted gene knockouts, with many such efforts using CRISPR/Cas9 editing reagents [10–13, 15, 16, 19–23]. Often, this process involves delivering a transgene to the plant genome that encodes the CRISPR guide RNAs (gRNAs) and Cas9 protein. Expression of these reagents in the T0 generation can generate mutation(s), which can be transmitted to subsequent generations. Moreover, the CRISPR/Cas9 transgene will in many instances not be linked to the mutation(s). Therefore, the breeder/geneticist can specifically select for segregating individuals in the subsequent generation that carry the desired mutated allele and no longer harbor the transgene.

In soybean, there are two main methods to create stable transgenic plants: *Agrobacterium*-based methods and biolistics. *Agrobacterium*-mediated transformation uses specific strains of either *Agrobacterium rhizogenes* or *A. tumerfacians* as a means to deliver a vector containing a transgenic DNA (T-DNA) cassette into the soybean host [24–27]. Biolistics is a direct gene transfer mechanism that uses high-velocity microprojectiles to introduce foreign DNA into tissues, resulting in non-homologous integration of transgenic DNA into the genome [28–34].

Soybean genes have been successfully modified using CRISPR/Cas approaches in both somatic and germline transmissible cells and for a variety of agronomic traits [35–42]. One recent study [42] carefully tracked the transmission of mutations and transgenes from T0 soybean plants to the next generation. In this study, *Agrobacterium* was used to transform CRISPR/Cas9 into whole soybean plants to knockout genes involved in small RNA pathways. Curtin et al. [42] targeted three genes, GmDrb2a, GmDrb2b and GmDcl3a and generated mutations at each target site in the T0 generation.

The GmDrb2 CRISPR construct used two guide-RNAs that each recognized both GmDrb2ba and GmDrb2b loci. The resulting transformation yielded two T0 plants derived from the same cluster of cells. From these two events, Curtin et al. [42] detected four small deletions at the GmDrb2a locus that were in common for both transgenic events. Screening of the GmDrb2b locus reveled two small deletions shared between the transgenic events and a 6 bp deletion unique to one of the T0 plants. Using next-generation sequencing, they identified three separate transgenic insertion events in the same locations for both T0 plants. After self-pollinating the T0 plants to the T1 generation, PCR screening for mutations revealed that only two of the four small deletions at GmDrb2a were transmissible. Similarly, only two of the three small deletions at the GmDrb2b locus were transmissible. Further analysis of each of the three transgenic insertions in the T1 revealed that each locus was transmissible.

Meanwhile, a different CRISPR/Cas9 construct was designed to target GmDcl3a [42]. Analysis of the GmDcl3a CRISPR mutations in two separate T0 plants identified a total of three different small deletions and one small insertion at the target site. PCR screening and next-generation sequencing of the T0 plants revealed a single transgenic insertion event in one of the plants and no evidence for transgenic insertion in the other (the latter of which was corroborated by sequence data). The authors then analyzed 60 T1 plants from each event and failed to identify any transmitted mutations or transgene integration events in either lineage.

The inconsistent transmission of mutations and transgenes observed among the soybean CRISPR/Cas9 lines in Curtin et al. [42] is based on a small number of plants/events. Therefore, in this study, we sought to expand upon this work by investigating more lines to identify expected and/or novel outcomes. We sequenced four T0 parents and four offspring of transgenic CRISPR/Cas9 lines to study the effects of CRISPR/Cas9 at gRNA target sites, as well as variation induced due to transgenic insertion events into the genome. The transformed lines studied in this experiment demonstrate a range of potential outcomes of CRISPR/Cas9 mutagenesis in soybean using an *Agrobacterium*-mediated transgenesis system.

## Results

### Identification of CRISPR mutations at target sites in T0 plants

**Fig. 1:**
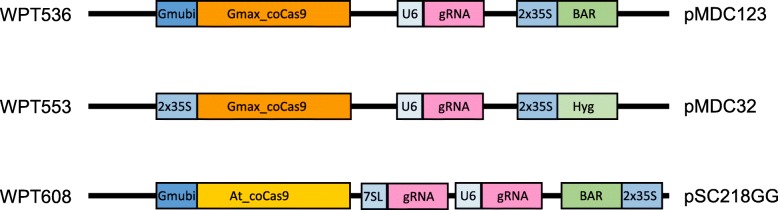
Transformation vectors used in whole-plant transformations. Plant expression cassettes were integrated into the host soybean genome through *Agrobacterium*-based whole-plant transformation methods using destination vectors listed on the right hand side. Promoters are blue, Cas9 endonucleases are orange, plant-selectable markers are green, and guide RNAs are pink. Different shading within each color group indicates different variants for each sequence class (e.g., the GmUbi and 35S promoters are different shades of blue)

Three separate whole-plant transformation (WPT) series named WPT536, WPT553, and WPT608 were generated using the expression vectors diagramed in Fig. 1. Each vector used a constitutive promoter (Gmubi or Cauliflower mosaic double 35S [43, 44]), a Cas9 endonucleases (Soybean codon optimized [36] or *Arabidopsis thaliana* codon optimized [45]), single or double gRNA cassette [42] driven either by the *A. thaliana* U6 or 7sL promoter, and a gene encoding resistance to either Glufosinate (BAR) or Hygromycin (Fig. 1, in Additional file 1: Table S1). Guide-RNA cassettes were constructed and inserted into each WPT destination vector. WPT536 and WPT553 each targeted a single locus on one gene model, Glyma.16 g090700, and Glyma.18 g041100, respectively (Table 1). WPT608 included two gRNAs targeting gene model Glyma.16G209100. One of these gRNAs had a perfect match to the target site on Glyma.16G209100 and nearly a perfect match to its paralog gene model Glyma.09G159900 (it had a 1 bp mismatch 16 bp from the PAM site). The other gRNA for gene model Glyma.16G209100 failed to result in mutations and is not further discussed below. Each destination vector was transformed into the background Bert-MN-01, and DNA was extracted from putatively transformed T0 plants.

**Table 1:** Mutation profiles induced by CRISPR/Cas9 and number of tragene insertions for each transgenic series

Plant number	Transgene integration	Target gene(s)	Target 1	Target 2
536–2	Chr11	Glyma.16 g090700	∆2-bp, + 1-bp	NA
536–2–13–15	NA	Glyma.16 g090700	No mutations	NA
536–2–13–16	NA	Glyma.16 g090700	∆2-bp	NA
553–6	NA	Glyma.18 g041100	∆7-bp, ∆7-bp	NA
553–6-8	NA	Glyma.18 g041100	∆2-bp	NA
553–6-11	NA	Glyma.18 g041100	∆6-bp	NA
608–1	Chr17	Glyma.16G209100, Glyma.09G159900	∆4-bp, ∆4-bp, + 1-bp	∆4-bp
608–3	Chr06, 09, 16, 18	Glyma.16G209100, Glyma.09G159900	∆3-bp, + 1-bp, TGI^a^	TGI^a^

^a^*TGI* Transgene integration at CRISPR/Cas9 target site

PCR-based gel assays (as described in [42]) were used to screen for mutations at the intended sites for each T0 plant. Four T0 plants were identified with putative mutations, one each from the WPT536 (individual WPT536–2) and WPT553 (individual WPT553–6) series, and two from the WPT608 series (individuals WPT608–1 and WPT608–3). Sequencing of PCR amplicons at each of the target sites for these four T0 plants revealed mutations (details are provided in the sections below). These four plants and some of their progeny were tracked for the inheritance of the targeted mutations and transgene integration loci.

### WPT536–2: expected transmission and segregation patterns from single transgene and mutation events

**Fig. 2:**
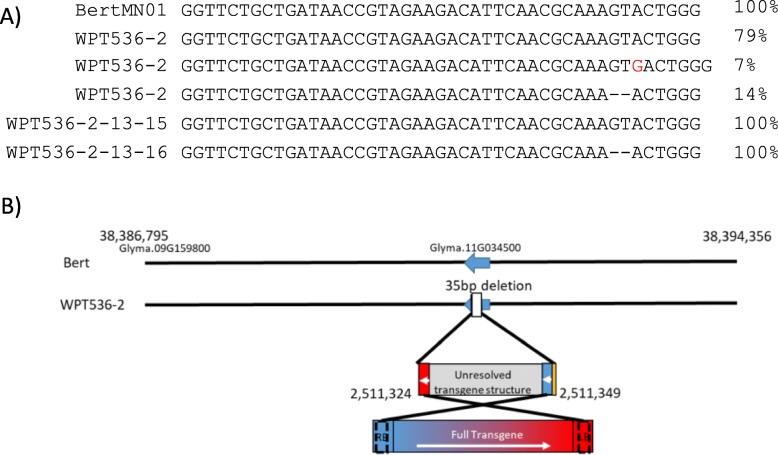
Whole-genome sequencing results of the transgenic series targeting GmRin4b. **a**) The sequences of transgenic plants and the Bert-MN-01 control at gRNA target site are shown. Sequences labeled ‘WPT536–2’ are from the T0/M0 plant while the bottom two sequences are from the M2 progeny. Dashes represent a deletion within a sequence, while red text indicates an insertion. Percentages represent the proportion of reads in a given sample showing each respective sequence. **b**) The diagram depicts WGS detection of the transgene insertion event and the variation induced at the insertion site. The blue to red gradient represents a color map of a transgene cassette to associate which segments of the transgene are integrated into the genome. The colors flanking the unresolved transgene structure are integration sites identified through sequencing associate to areas of the transgene colormap with orange representing an addition

WPT536–2 was a T0 plant transformed with a Gmubi-driven *Glycine max* codon-optimized Cas9 and a single gRNA targeting Glyma.16 g090700 (herein known as GmRin4b). PCR confirmed the presence of the Cas9 and plant-selectable marker (in Additional file 2: Fig. S1), indicating successful transformation of the construct. Sequencing of a PCR amplicon from the gRNA target site revealed a 2 bp deletion. Whole genome sequencing (WGS) of the T0 plant confirmed the previously identified 2 bp deletion along with evidence of a 1 bp insertion at the target site (Fig. 2 a, in Additional file 2: Fig. S2).

Furthermore, WGS revealed a single CRISPR/Cas9 transgene integration site localized to an interval on chromosome 11 (Fig. 2b, in Additional file 2: Fig. S3). The interval had a 35 bp hemizygous deletion and a 4 bp addition flanking one side of the transgenic insertion (Table 2). The reads spanning the genome into the transgene appear to suggest a complete cassette between the right border (RB) to left border (LB) was inserted within the deleted region. Given the presence of both a transgene and mutation, the generation of this plant was renamed T0/M0.

**Table 2:** Types of variation induced for each transgene insertion event

Plant number	Transgene integration genome position	Type	Genic	Base pair addition size surrounding transgene insertion
536–2	Chr11: 2,511,324-2,511,349	∆35-bp	Yes	4 bp, 0 bp
608–1	Chr17: 37,687,748	∆1-bp	No	unknown, 9 bp
608–3	Chr06: 3,498,485-3,498,492	∆8 bp	Yes	3 bp, 20 bp
608–3	Chr09: 38,390,575-38,390,586	∆10-bp ^a^	Yes	0 bp, 11 bp
608–3	Chr16: 36,848,517	∆1-bp ^a^	Yes	0 bp, 0 bp
608–3	Chr18: 55,616,603–55,616,607	∆3-bp	No	0 bp, 0 bp

^a^Transgene integration at CRISPR/Cas9 target site

PCR screening of GmRin4b mutations in the segregating T1/M1 and T2/M2 generations revealed germline transmission of the transgene. However, these assays revealed that four out of 27 T1/M1 plants no longer carried the transgene (such plants can simply be identified as M1 progeny, as they do not have the transgene). Confirmation of this result for the M1 plant WPT536–2-13 and its M2 progeny is shown in Additional file 2: Fig. S1. WGS was performed on two M2 progeny from WPT536–2-13 (plants WPT536–2–13–15 and WPT536–2–13–16). To further validate that there was no trace of transgenic DNA, reads from WGS were mapped directly to the transgene for each sequenced plant (in Additional file 2: Fig. S4). Only the T0 parent had consistent coverage across the transgene, while the progeny plants lacked any reads mapping to the transgene except for the Gmubi promoter, which can be attributed to the natural ubiquitin promoter sequences located in the soybean genome. Furthermore, the WGS revealed that the M2 plant WPT536–2–13–16 retained the 2 bp mutation at the CRISPR target site while plant WPT536–2–13–15 segregated back to homozygosity for the wild-type allele. Given these findings, it was determined that plant WPT536–2–13–16 is a simple M2 generation plant (contains a mutation, but no transgene), while plant WPT536–2–13–15 is neither a transgenic or mutant individual. This segregation represents expected Mendelian outcomes, wherein the respective transgenic and mutated loci could be selected for or against in subsequent generations.

### WPT608–1: T0 transgenes and mutations were not transmitted to progeny

**Fig. 3:**
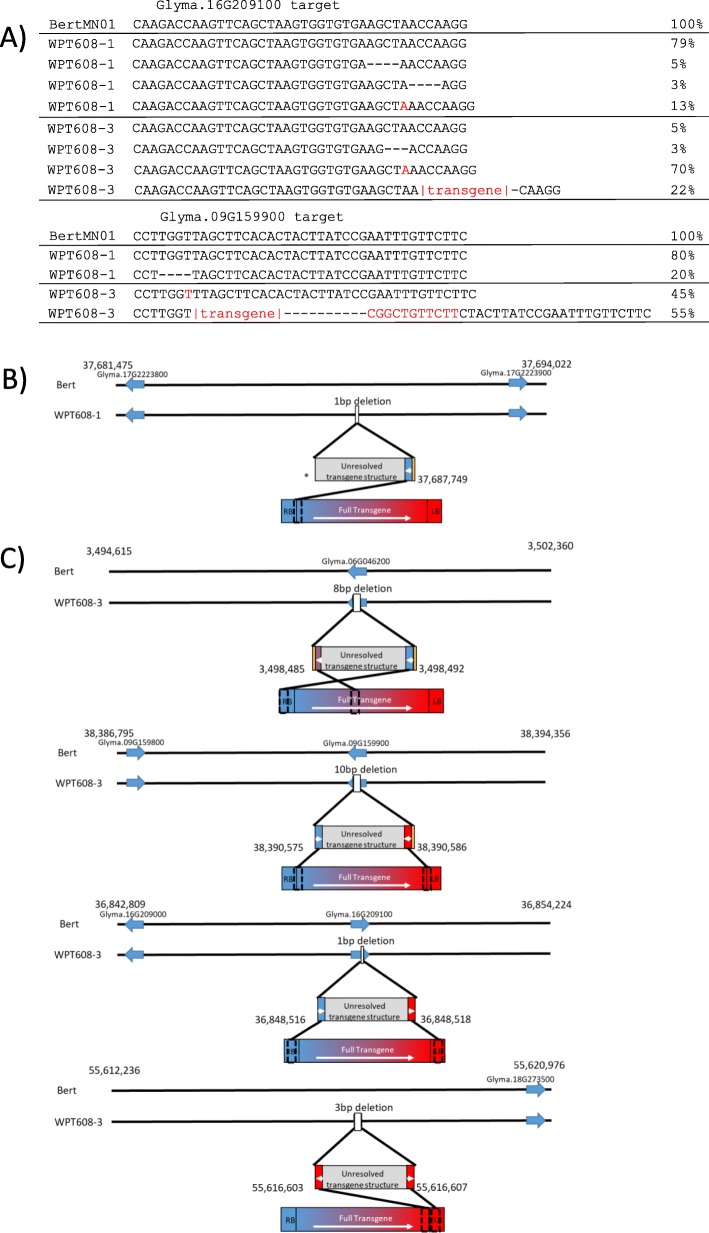
Screening of mutations and transgene insertions in the transgenic series WPT608–1 and WPT608–3, targeting Glyma.16G209100 and Glyma.09G159900. **a**) The sequences at the gRNA target sites in the T0 plants compared to the Bert-MN-01 control. Dashes represent a deletion within a sequence, red text represents insertions. Percentages indicate the proportion of reads for each of the mutations in the respective T0 plants. The Glyma.16G209100 sequence is shown in the sense orientation while the Glyma.09G159900 sequence is shown in the antisense orientation. **b**) The diagram depicts WGS detection of the transgene insertion locus and the variation induced at the insertion site for WPT608–1. Gold represents additions distinct from transgene and reference. The asterisk represents an unresolvable junction due to low sequencing coverage. **c**) The diagrams depict WGS detection of the transgene insertion loci and the variation induced at the four insertion sites for WPT608–3. Gold represents additions distinct from transgene and reference

Gene models Glyma.16G209100 and Glyma.09G159900 were targeted by CRISPR/Cas9 using a construct nearly identical to that used by Curtin et al. [42], with the only modification being the gRNA target site. PCR screening revealed that two lines, WPT608–1, and WPT608–3, had evidence for mutations at recognition sites on chromosomes 9 and 16 from a single gRNA, as well as evidence of transgene integrations into the genome. WGS of 608–1 confirmed the presence of a 1 bp insertion and two different 4 bp deletions as seen by PCR (Fig. 3a). Furthermore, an additional target site on the paralogous gene model Glyma.09G159900, which has an identical gRNA recognition site, also showed evidence for mutation, as 20% of the T0 reads had a 4 bp deletion at the target site (Fig. 3a).

WGS identified a single transgene integration site on chromosome 17 for WPT608–1 (Fig. 3b). The T-DNA segment induced a 1 bp deletion at the transgene integration site with a 9 bp insertion flanking the transgenic segment (Table 2). Reads that spanned the genomic-transgene junction revealed that a portion of the right border inserted itself into that location. The transgenic sequences at the left junction were undetectable due to the lack of any chimeric reads aligning to that segment of the genome.

PCR assays could not detect any presence of mutations or the transgene in the T1/M1 generation among 22 tested plants, suggesting that neither the mutations nor the transgenic insertion event were germline transmissible from WPT608–1 (in Additional file 2: Fig. S5). Therefore, the WPT608–1 event appears to most likely be an instance where the T0 plant was chimeric, and the transgenic/mutated sector did not produce seeds. Alternative hypotheses may also explain this outcome, such as the transgenic and mutated sequences originated from different sectors, with the mutations being driven by transient expression of the reagents. In any case, mutations appear to have been produced in some somatic cells of the T0 plant but did not reach the germline.

### WPT608–3: mutations and transgene integrations at the CRISPR target sites

WGS of 608–3 revealed four separate transgene insertion events on chromosomes 6, 9, 16, and 18 (Fig. 3c, in Additional file 2: Fig. S6). The event on chromosome 6 induced an 8 bp deletion in the host genome while inserting 3 and 20 bp additions on either side of the transgene integration site (Table 2). Analysis of the reads spanning the genomic/transgene junctions suggests that there was a partial insert of half of the transgene from the RB to halfway through the cassette. The transgenic insertion event on chromosome 18 deleted 3 bp of the host genome and created a more complex transgenic insertion event. The transgenic sequence detected on the left junction was in the antisense orientation while the sequence on the right junction was in the sense orientation, suggesting that there were multiple insertions/rearrangements of the transgene at that location (Fig. 3c).

The transgene integration site on chromosome 16 was observed within the CRISPR gRNA target site on gene model Glyma.16G209100 (in Additional file 2: Fig. S7). The sequenced regions flanking the transgene integration site indicated that 1 bp of the host genome was deleted while inserting a full transgene cassette. Furthermore, the transgene integration site on chromosome 9 was also observed within a CRISPR gRNA target site on the paralogous gene model Glyma.09G159900 (in Additional file 2: Fig. S8), and that it created a 10 bp deletion in the host genome. There was also a 11 bp insertion flanking the sequence of one end of the chromosome 9 transgene integration site (Table 2). Reads spanning the junctions of both the chromosome 9 and chromosome 16 events suggest that a full transgene cassette was inserted into both locations.

All six of the tested WPT608–3 T1 progeny showed inheritance of the transgene integration event at the Glyma.16G209100 locus (in Additional file 2: Fig. S6). Two of the six progeny (plants WPT608–3-2 and WPT608–3-3) were homozygous for this transgene integration event (in Additional file 2: Figs. S6 and S9). PCR and sequencing assays for two other WPT608–3 T1/M1 progeny (plants WPT608–3-1 and WPT608–3-5) confirmed germline transmission of the 1 bp insertion allele at Glyma.16G209100 (in Additional file 2: Fig. S9). Meanwhile, the transgene insertion at the paralogous locus Glyma.09G159900 was only inherited by four of the six progeny, and none were homozygous for this event (in Additional file 2: Fig. S6). Furthermore, all six of these plants showed evidence for inheriting the 1 bp insertion allele at Glyma.09G159900 (in Additional file 2: Fig. S9).

In summary, WPT608–3 represents a unique T0 plant in which two of the four transgene integration sites were located at the gRNA target site. Presumably, this was caused by CRISPR/Cas9 induction of double-stranded breaks at the paralogous target sites that were repaired by transgene integration during the transformation process.

### WPT553–6: unresolved transgene inheritance in a line with germline mutations

**Fig. 4:**
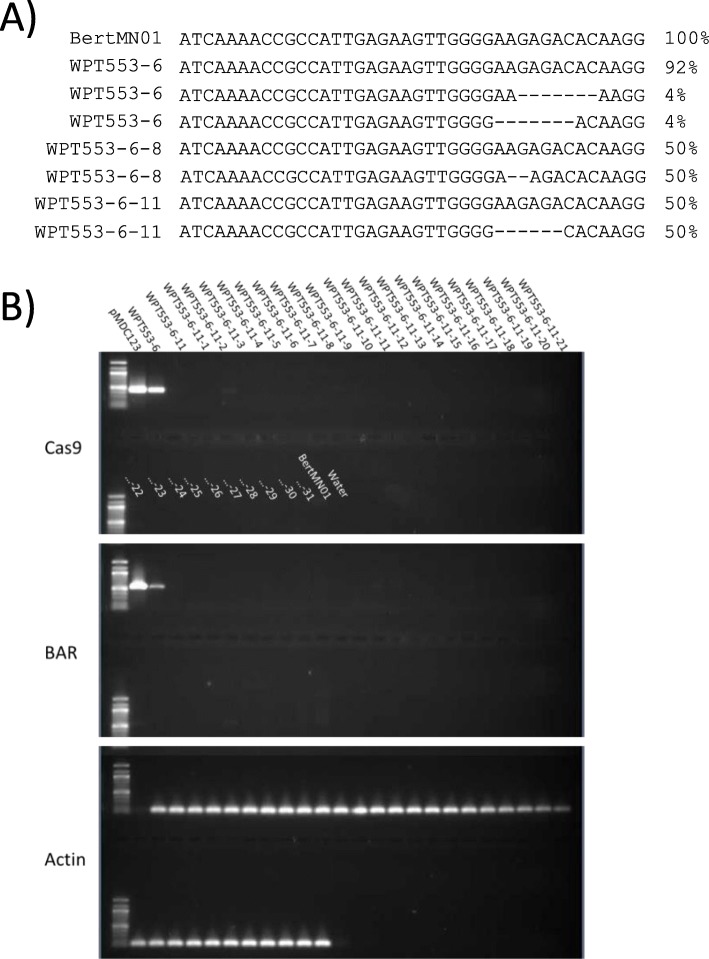
Screening of markers and mutations in the transgenic series targeting Glyma.18 g041100. **a**) The sequences at the gRNA target site for the transgenic plants and Bert-MN-01 control. Sequences from the T0 plant are labeled as WPT553–6 and the lower sequences (labeled WPT553–6-8 and WPT553–6-11) are from the T1 progeny. Dashes represent a deletion within a sequence. Percentages indicate the proportion of reads for each of the mutations in the respective plants. **b**) PCR assay screening for presence/absence of the Cas9 endonuclease, BAR plant-selectable marker, and actin control. Lane are labeled as the transformation vector control (top row and far left for each assay), the T0 plant (WTP553–6), the subsequent generation (WPT553–6-11), and the following generation (WPT553–6-11-x plants)

The CRISPR/Cas9 construct targeting Glyma.18 g041100 (herein known as GS1) was developed as a result of a previous study and shown to be effective at generating mutations in soybean somatic hairy root tissues [36]. We used the same construct in whole-plant transformation to generate the WPT553 series of plants for the present study. PCR screening and WGS of the WPT553–6 T0/M0 plant revealed the presence of the transgenic sequence and two different 7 bp deletions at the target site (Fig. 4a). Sequencing of the progeny plants 553–6-8 and 553–6-11 identified a 2 bp and a 6 bp mutation in the respective plants. Neither of these mutated alleles were identified in the T0/M0 parental plant (in Additional file 2: Fig. S10). Furthermore, the plant-selectable marker and the Cas9 were not detected by PCR in the 553–6-8 and 553–6-11 plants, nor were these transgene components detected in any of the 31 putative T2/M2 offspring (Fig. 4b). Aside from the 553–6-8 and 553–6-11 individuals, none of these plants showed evidence for mutations at the target site.

To help detect chimeric transgenic or mutation events, leaf tissue was pooled from different parts of plant WPT553–6, and DNA was prepared for WGS. Similar pooling strategies were also applied within each of the 553–6-8 and 553–6-11 offspring plants. Despite the PCR evidence indicating the presence of transgenic sequences (Fig. 4a), WGS analyses were not able to identify any transgene integration sites in the WPT553–6 T0 plant. Furthermore, no such integrations sites were identified in the 553–6-8 and 553–6-11 offspring. When mapping the DNA of each plant directly to the transgene (in Additional file 2: Fig. S11) only the WPT553–6 T0 plant had reads that consistently mapped to the transgene. However, the average read coverage for the transgene was far below the WPT plants described in previous sections that exhibited heritable transgenic insertion events. WGS mapping of reads to the transgene sequence for 553–6-8 and 553–6-11 respectively yielded only 7 and 1 reads that mapped (in Additional file 2: Fig. S11). Therefore, the extremely low mapping coverage to transgenic sequences observed in the WPT553 plants may be better explained by trace levels of sample contamination rather than the presence of a stably integrated transgene or due to cross-contamination due to template switching within barcoded libraries [46]. Therefore, we speculate that the initial mutagenesis observed in the WPT553–6 T0 plant may have been derived from a non-integrated CRISPR/Cas9 transgene, which may explain the transmission of mutated alleles with minimal evidence for transmission of any transgene components.

### Sequence microhomology near transgenic insertions sites

**Fig. 5:**
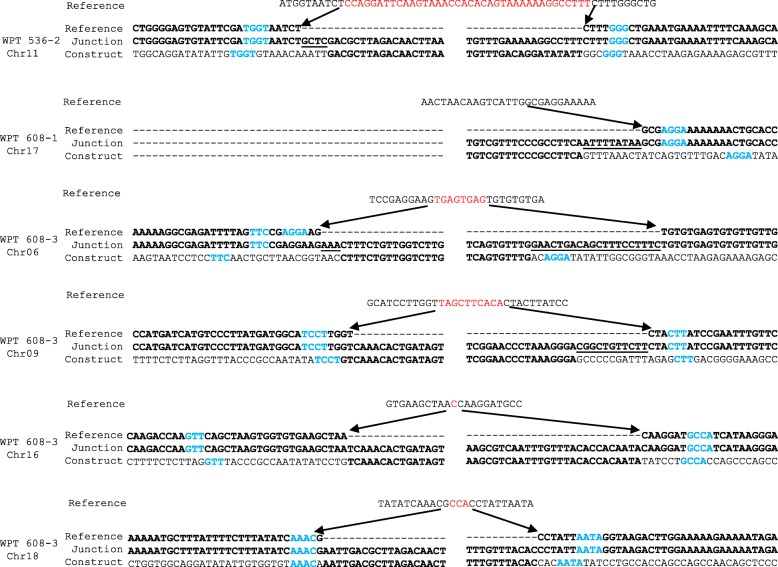
Microhomology evidence at transgene integration sites. Whole genome sequencing results at transgene insertion sites. Bases in blue represent regions of microhomology between the construct and reference genome. Bases in red represent the sequences deleted from the host genome at the transgene insertion. Bold sequences represent the sequences remaining in the transgenic plant. Underlines indicate base additions at the transgene insertion sites not previously found in the host genome nor the construct

Analyses of the transgenic integration sites revealed evidence of sequence micro-homologies between the inserted transgenic DNA and the host sequences flanking the insertion. We aligned the putative host genome sequence (based on the Williams 82 reference genome), the transgene construct sequence, and the observed sequence at the transgene integration junction to identify potential sites of microhomology (Fig. 5). The junction for the T-DNA insertion typically exhibited sequence matches for three or four base-pair (bp) tracts in regions flanking the transgene integration site. For instance, the chromosome 18 integration site in plant 608–3 had a perfect match in homology between the construct and the host genome sequence in the region flanking the 5′ end of the insertion, while the microhomology on the 3′ end was shifted three bp between the host genome and transgenic sequence (Fig. 5). While the 5′ junction of 608–3 on chromosome 18 was the only instance of a perfect micro-homology match, 8 out of the 11 junctions that were detected were within 3 bp of one another, while 2 of the 11 were within 9 bp of one another. Interestingly, each microhomology sequence across all 11 junctions contained a homopolymer sequence of at least 2 bp.

## Discussion

Resequencing of four T0 plants and selected progeny provided a high-resolution view of transgene integration structures and gene editing events. The four T0 plants each exhibited a different outcome, though each outcome parallels similar findings in the recent crop genome editing literature. Plant WPT536–2 exhibited the most straight-forward scenario, in which a single transgene integration produced frameshift mutations at a single target site. The transgene and mutations transmitted and segregated in the progeny, as is generally the desired outcome for the majority of such experiments and has often been reported in previous studies [41, 42, 47–56].

Plant WPT608–1 exhibited evidence for a single transgene integration and targeted mutations at two paralogous loci. However, neither the transgenes nor mutations were recovered in the progeny. This type of negative result may be commonplace in genome editing projects, but it is an undesirable outcome for most projects and is likely to be unreported in scientific articles [42]. There are different mechanisms that may explain this result, including the possibility that WPT608–1 was a chimeric plant in which the transgene and mutations were part of a sector that did not produce seeds. It is noteworthy that the DNA used to resequence plant WPT608–1 was pooled from five different leaflets growing on different branches of the plant. Perhaps only one or two branches harbored the transgene and mutations, and these failed to produce seeds. Probably the simplest explanation would be that somatic mutations were identified in the T0, however by chance and circumstance, none of the meristems that eventually produced offspring harbored such mutations. While this hypothesis remain untested, there are additional speculations and hypotheses that could be suggested to explain the observed result.

Plant WPT608–3 exhibited an unexpected phenomenon in which two paralogous CRISPR target sites were each found to harbor CRISPR/Cas9 transgenes. The process to create such loci is somewhat analogous to a previously described non-homologous end-joining strategy used to insert a specific T-DNA segment into a specific genomic locus [57]. In this strategy, the editing reagent (e.g., the CRISPR/Cas9) is designed to simultaneously cut both the intended T-DNA segment from the transgene and the genomic target where the T-DNA is to be inserted. In effect, the released T-DNA segment acts as a donor molecule that can be integrated into the genomic target site during the double-stranded break repair. In the case of plant WPT608–3, it appears that when the full transgene was delivered to the cell it generated double-stranded breaks at the intended paralogous loci, and then copies of the transgene were used to repair the targeted double-stranded breaks. Site specific T-DNA integration in plants have been previously reported in the literature [38, 58–62], though it is not common and we are unaware of any examples in which two unlinked (in this case, paralogous) target sites acted as transgene integration loci in a single cell. Importantly, all four transgenic loci in the T0 plant were shown to segregate in subsequent generations. Furthermore, a simple frameshift allele for gene model Glyma.16G209100 was also shown to segregate in these generations. Therefore, a researcher could select for progeny that specifically carry the frameshift allele and no longer harbor the transgenes, if such an outcome is desired.

Plant WPT553–6 exhibited a unique outcome in which the T0 plant exhibited the presence of mutations at the targeted locus (Glyma.18 g041100), however resequencing data could not confirm integration of the CRISPR/Cas9 transgene. Analysis of progeny indicated that a small number of plants (two out of 31) carried mutations, while none of the plants harbored the transgene. On the surface, this appears to be a highly favorable outcome, as transmissible mutations were recovered in an apparently non-transgenic background. However, this may be a difficult result to reproduce, as it would seem to require transient expression of the transgene without integrating into the host genome, thereby generating mutations in a non-transgenic background. Zhang et al. [63] reported a purposeful identification of such plants in wheat, wherein the authors specifically screened plants bombarded with CRISPR/Cas9 constructs for individuals carrying mutations and no transgenes [63]. This process was able to identify plants of this type but required extensive screening of large populations to identify these rare events. In the case of WPT553–6, it is also possible that the transgene did insert stably into the genome, but was located in a region of the genome difficult to map and/or a structurally rearranged T-DNA was inserted such that it was not detected by PCR or resequencing. Alternatively, as discussed for WPT608–1 above, it is possible that the WPT553–6 transgene integration may have disrupted a critical process for gametophyte or early sporophyte survival and was thus not able to be recovered in the progeny. This would not entirely explain the inability to identify the transgene integration site in the T0 plant, but would provide an explanation for the failure to transmit the transgene to progeny.

Regardless of the construct used in each whole-plant transformed line, each junction displayed evidence of microhomology between the reference genome and the transformation vector. While the distribution of integration sites is spread throughout the genome, the evidence of microhomology flanking each of the transgene integration sites further reinforces that this process is not entirely random [64].

Despite the complications of working with these complex plants, there is a high probability to recover a desired product using the CRISPR/Cas9 technology in soybean. In this study, we used two different Cas9 endonucleases, and they yielded similar mutation profiles between events. While the size of mutations observed were all under 7 bp in size, all but one mutation induced at a gRNA target site created a frame-shift mutation, most likely knocking out the function of the target gene. In the case of multiple transgene insertions, it may be difficult to completely segregate away all the transgenic copies in subsequent generations. However, additional backcrosses or outcrosses can be used to remove these loci, as demonstrated by Curtin et al. [42]. This is a relatively minor inconvenience, given the capacity to generate vast and novel allelic diversity for so many loci.

## Conclusions

The results described in this study highlight the range of outcomes one might expect from strategies that rely on stable transformation of a DNA editing construct. Such experiments can be complicated, as they typically require a minimum of two loci of interest, the transgene integration site(s) and the targeted region(s). This quickly becomes more complex when there are multiple unlinked transgene integrations and when there are multiple gene editing targets. Furthermore, unexpected segregation patterns may be driven by several potential mechanisms, such as plant chimeras, editing of intended and paralogous targets, linkage between the transgene integration and target site, and transient expression of the editing reagents without transgene integration into the host genome. Genome resequencing provides high-resolution of transgene structures and editing events, enabling researchers to diagnose both the expected and unexpected segregation outcomes from these lineages.

## Methods

### Generation of whole plant transformant expression vectors

Plant expression vectors were created using three different binary vectors; PMDC123, PMDC32, and pNB96 [2, 65]. The expression vector used to create WPT536 was a modified version of the Cas9 MDC123 found on addgene.org (https://www.addgene.org/59184/). The vector was modified by replacing the 2x35S Cas9 promoter with a *Glycine max* ubiquitin promotor [44] and adding the Rin4b (Glyma.16 g090700) gRNA recognition sites. The WPT553 expression vector, MDC32/GUS/GmCas9, was originally developed and used in a previous publication [36]. WPT 608–1 and 608–3 used the same pSC218GG construct used in previous work [42], except with different gRNA recognitions sites for the Glyma.16G209100 (and Glyma.09G159900) target sites.

### Identification of CRISPR/Cas9 target sites

CRISPR target sites were identified using a soybean CRISPR design website (http://stuparcrispr.cfans.umn.edu/CRISPR/) [36]. Glyma numbers from the Wm82.a2.v1 soybean reference were used as input into the webtool, and target-sites were screened for unique restriction sites designed to cut 3–5 bp upstream of the proto-spacer adjacent motif.

### Delivery of expression vectors to soybean whole-plants

Constructs were delivered to the Bert-MN-01 background using 18r12, a disarmed k599 *Agrobacterium rhizogenes* strain [27]. Methods for delivery and growth of whole-plant transformants were performed as previously described [2].

### DNA extraction and identification of transgene insertion sites and mutation sites

Leaf tissue was harvested from five different soybean branches for each whole-plant transformant and extracted with a Qiagen DNeasy plant kit (item 69,106). DNA samples were sent to the University of Minnesota Genomics Center for sequencing using an Illumina HiSeq2500 with v4 chemistry to generate 125 bp parried-end reads. Sequencing was performed to approximately 20X genome coverage for each sample. Reads were checked for initial quality using Fastqc version 0.11.5 and Illumina Truseq adapters were trimmed using cutadapt version 1.8.1 with a minimum read length set to 40 bp and quality cutoff set to a phread score of 20 [66, 67]. To map reads to the soybean reference genome (Wm82.a2.v1), we used bwa version 0.7.12 with band width set to 100, mark shorter splits as secondary, and penalty for mismatch set to 6 [68]. Samtools version 1.6 was used to convert any SAM file format to BAM format, sort, and index files [69]. Identification of transgene insertion sites was performed in a manner similar to Srivastava et al. 2014 [70]. Fasta files were created using the transgene cassette with 100 bp flanking backbone sequence to serve as our reference genome. Sequenced reads were then mapped to the transgene reference using the same programs and parameters used to map reads to the reference genome. To detect transgene insertion junctions, reads that mapped to the transgene on only one of the two paired ends were extracted using a modified version of extract_unmapped_mates.pl from [70], to accept bam files as input. The other paired ends (those that did not map to the transgene and were termed orphan reads) were then mapped to the Wm82.a2.v1 reference using bowtie2 version 2.2.4 using -- local -- very-sensitive-local [71] to identify the genomic sequences adjacent to the transgene insertion. SAM files were then converted to BAM file format, sorted and indexed in the same manner mentioned above. Orphaned reads that mapped to the reference were further investigated upon using IGV version 2.3.90 [72]. Orphaned read mapping was then compared to read mapping to the soybean reference and the parental line (Bert-MN-01) as a control. Deletions were investigated using IGV at each CRISPR site throughout the genome. To automate this process, a custom bash script was created called TransGeneMap (https://github.com/MeeshCompBio/Soybean_Scripts) that allows users to input only the forward and reverse reads, index reference genome, and transgene sequence to automate the analysis.

Mutation analyses of CRISPR target sites were performed on T0 plants and progeny using PCR-based gel assays as previously described [42]. Sanger sequencing of PCR amplicons or cloned PCR products was used to identify and confirm specific mutations at these sites.

## Supplementary information

**Additional file 1: Table S1.** WPT construct metadata.

**Additional file 2: Fig. S1.** Screening of markers and mutations in the CRISPR transgenic series targeting Rin4b. **Fig. S2.** IGV screenshot of WGS at the gRNA target site for Rin4b.. **Fig. S3.** IGV screenshot of the CRISPR/Cas9 transgene (targeting Rin4b) insertion event using WGS. **Fig. S4.** Read mapping coverage for the transgene encoding the CRISPR/Cas9 targeting Rin4b. **Fig. S5.** PCR assays for transgene presence and targeted mutations on chromosome 16 and 09 for in WPT608–1 series. **Fig. S6.** Transgene detection in all offspring for WPT608–3. **Fig. S7.** IGV screenshot of Glyma.16G209100 gRNA target site and transgene insertion on chromosome 16. **Fig. S8.** IGV screenshot of the Glyma.16G209100 paralog (Glyma.09G159900) gRNA target site and transgene insertion on chromosome 9. **Fig. S9.** Analysis of the CRISPR target site on chromosome 16 and 09 for series WPT608–3. **Fig. S10.** GS1 (Glyma.18 g041100) mutations at the gRNA target site induced by CRISPR/Cas9. **Fig. S11.** Read mapping coverage for the transgene encoding the CRISPR/Cas9 targeting GS1.

## Data Availability

Sequencing data for all the samples in this study are deposited in the Sequence Read Archive (http://www.ncbi.nlm.nih.gov/sra/) under accession number PRJNA531962. Scripts used to run the analysis can be found at https://github.com/MeeshCompBio/WPT_analysis.

## Abbreviations

BAR: Glufosinate
bp: base-pair
CRISPR: Clustered Regularly Interspaced Short Palindromic Repeats
DSB: Double-stranded break
Gmubi: *Glycine max* ubiquitin
gRNA: guide RNA
LB: Left border
RB: Right border
T-DNA: Transgenic DNA
WGS: Whole genome sequencing
WPT: Whole-plant transformation
