# Supplementary material for: Integration, abundance, and transmission of mutations and transgenes in a series of CRISPR/Cas9 soybean lines
Source: BMC Biotechnol. 2020 Feb 24;20:10. doi: 10.1186/s12896-020-00604-3 (PMC7038615; doi:10.1186/s12896-020-00604-3)
Supplement: Supplementary file 2 — Additional file 2: Fig. S1. Screening of markers and mutations in the CRISPR transgenic series targeting Rin4b. Fig. S2. IGV screenshot of WGS at the gRNA target site for Rin4b.. Fig. S3. IGV screenshot of the CRISPR/Cas9 transgene (targeting Rin4b) insertion event using WGS. Fig. S4. Read mapping coverage for the transgene encoding the CRISPR/Cas9 targeting Rin4b. Fig. S5. PCR assays for transgene presence and targeted mutations on chromosome 16 and 09 for in WPT608–1 series. Fig. S6. Transgene detection in all offspring for WPT608–3. Fig. S7. IGV screenshot of Glyma.16G209100 gRNA target site and transgene insertion on chromosome 16. Fig. S8. IGV screenshot of the Glyma.16G209100 paralog (Glyma.09G159900) gRNA target site and transgene insertion on chromosome 9. Fig. S9. Analysis of the CRISPR target site on chromosome 16 and 09 for series WPT608–3. Fig. S10. GS1 (Glyma.18 g041100) mutations at the gRNA target site induced by CRISPR/Cas9. Fig. S11. Read mapping coverage for the transgene encoding the CRISPR/Cas9 targeting GS1. [file 12896_2020_604_MOESM2_ESM.pptx]

## Slide 1
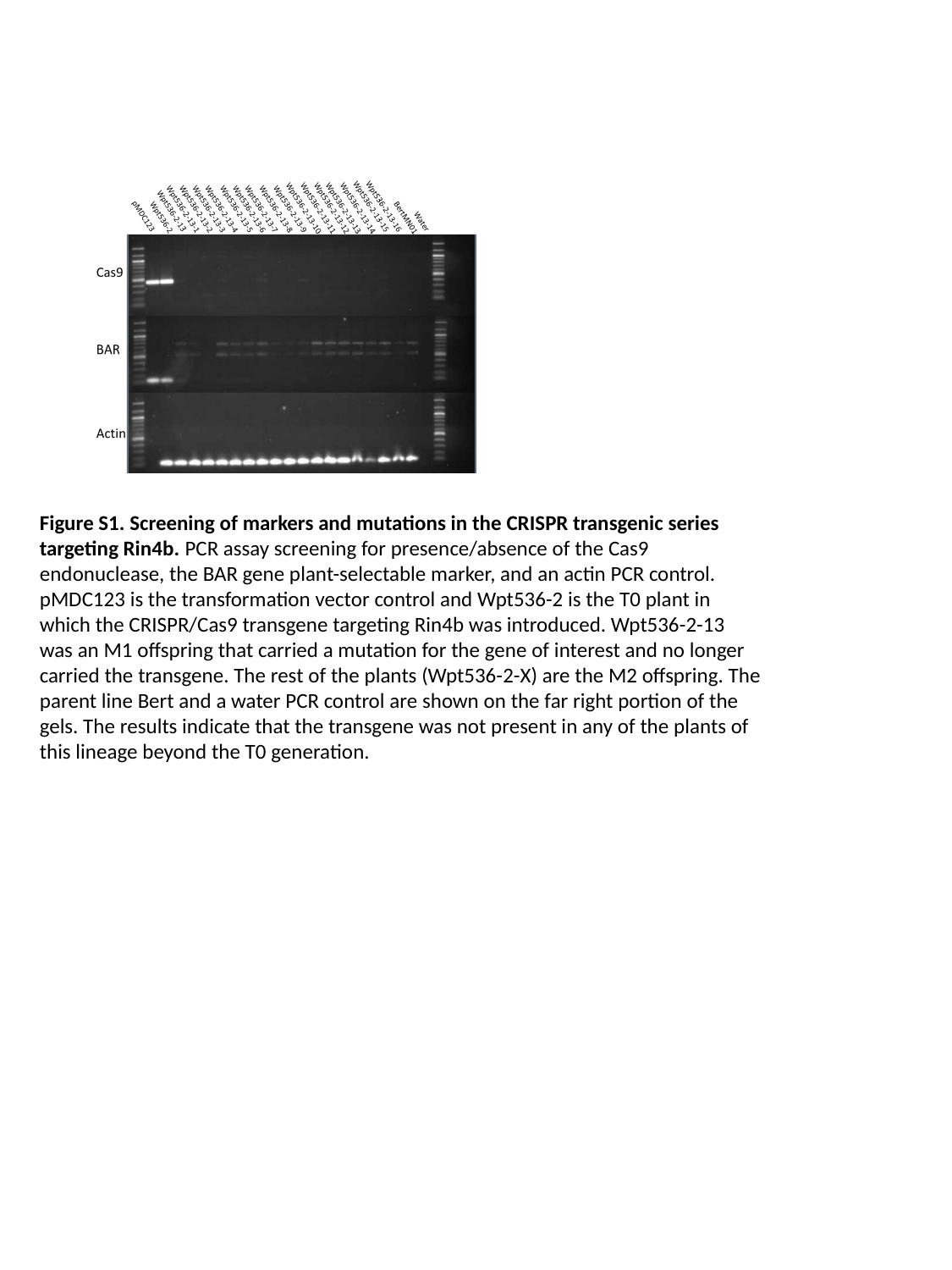

Figure S1. Screening of markers and mutations in the CRISPR transgenic series targeting Rin4b. PCR assay screening for presence/absence of the Cas9 endonuclease, the BAR gene plant-selectable marker, and an actin PCR control. pMDC123 is the transformation vector control and Wpt536-2 is the T0 plant in which the CRISPR/Cas9 transgene targeting Rin4b was introduced. Wpt536-2-13 was an M1 offspring that carried a mutation for the gene of interest and no longer carried the transgene. The rest of the plants (Wpt536-2-X) are the M2 offspring. The parent line Bert and a water PCR control are shown on the far right portion of the gels. The results indicate that the transgene was not present in any of the plants of this lineage beyond the T0 generation.

## Slide 2
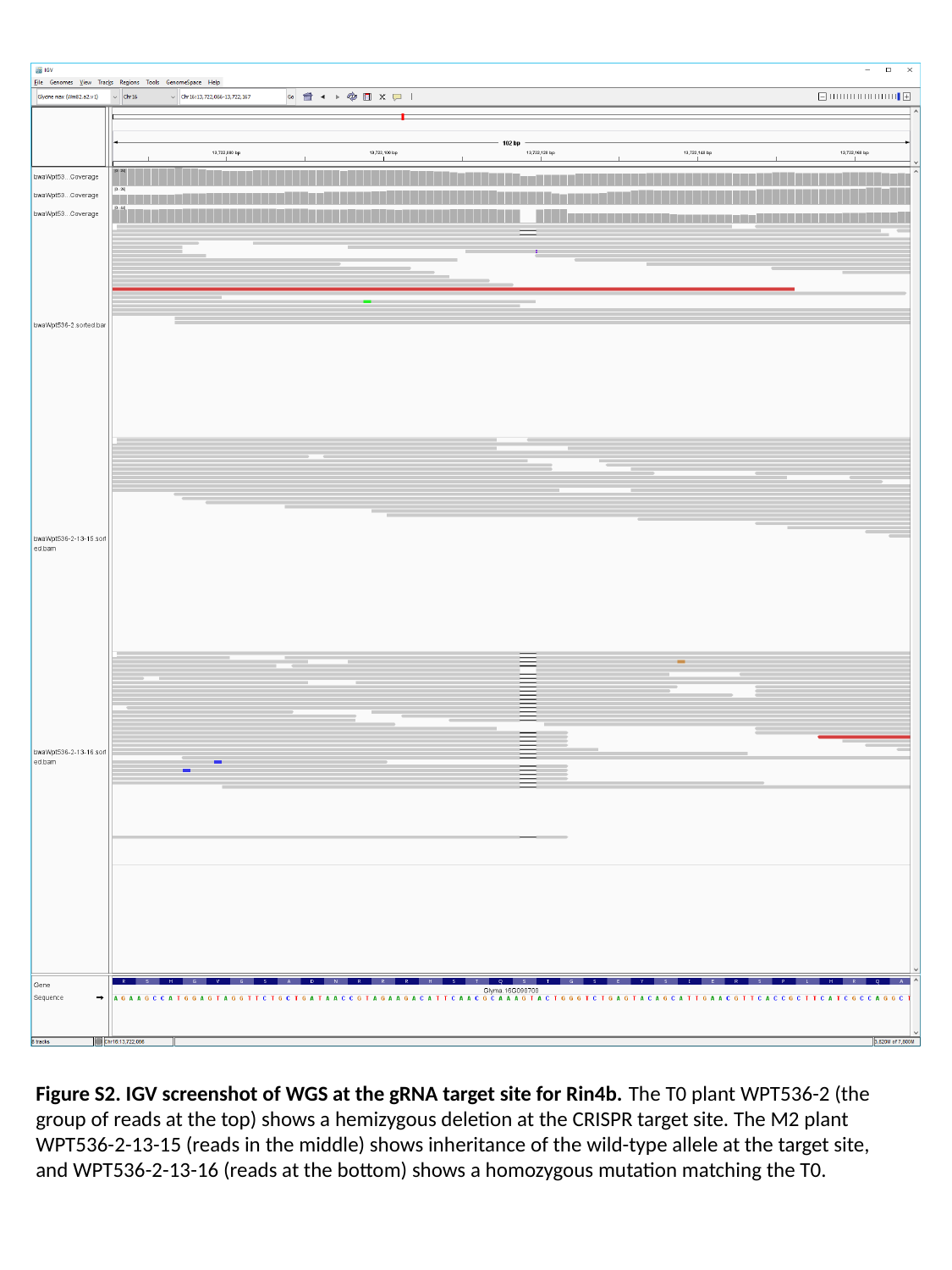

Figure S2. IGV screenshot of WGS at the gRNA target site for Rin4b. The T0 plant WPT536-2 (the group of reads at the top) shows a hemizygous deletion at the CRISPR target site. The M2 plant WPT536-2-13-15 (reads in the middle) shows inheritance of the wild-type allele at the target site, and WPT536-2-13-16 (reads at the bottom) shows a homozygous mutation matching the T0.

## Slide 3
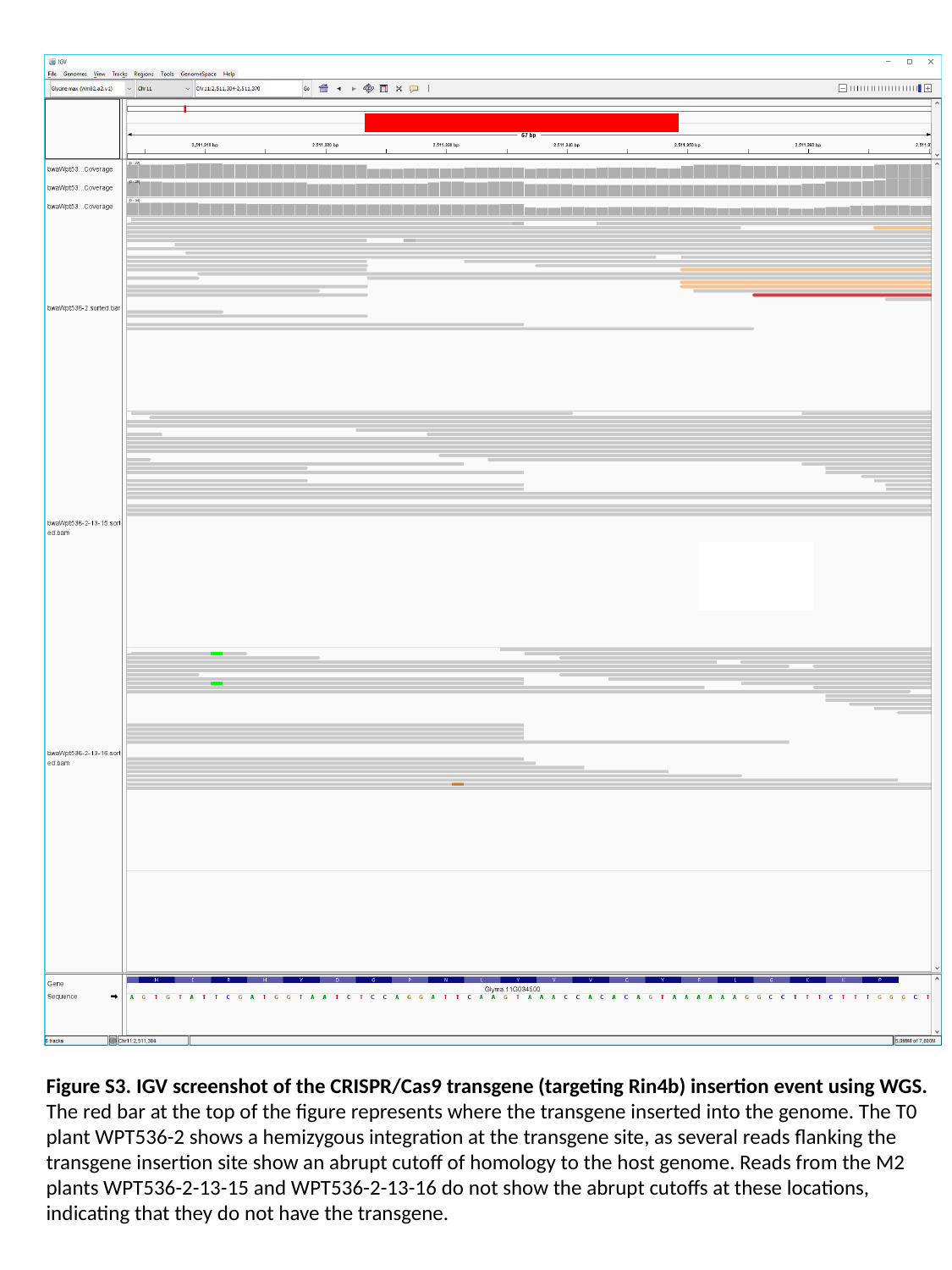

Figure S3. IGV screenshot of the CRISPR/Cas9 transgene (targeting Rin4b) insertion event using WGS. The red bar at the top of the figure represents where the transgene inserted into the genome. The T0 plant WPT536-2 shows a hemizygous integration at the transgene site, as several reads flanking the transgene insertion site show an abrupt cutoff of homology to the host genome. Reads from the M2 plants WPT536-2-13-15 and WPT536-2-13-16 do not show the abrupt cutoffs at these locations, indicating that they do not have the transgene.

## Slide 4
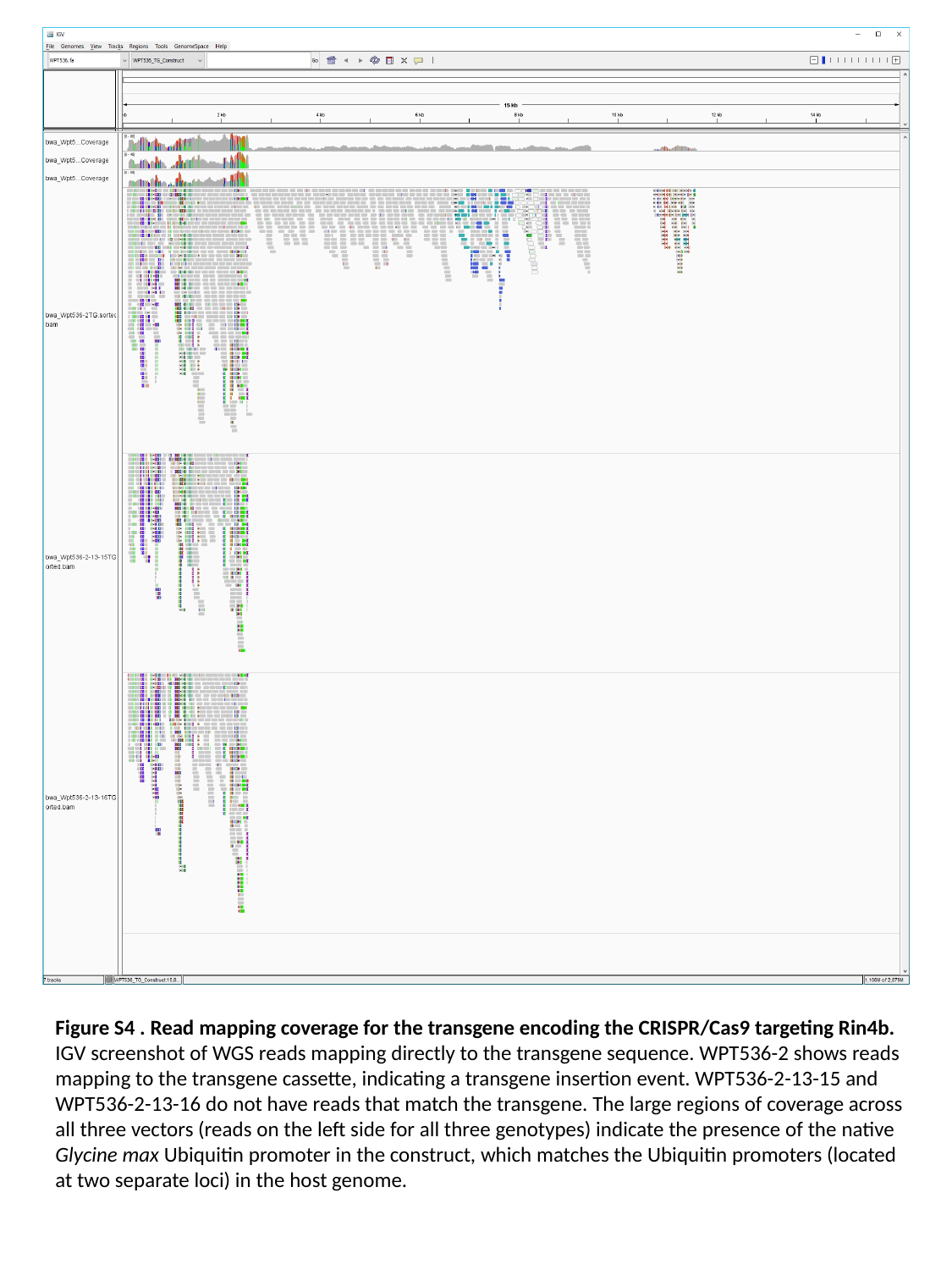

Figure S4 . Read mapping coverage for the transgene encoding the CRISPR/Cas9 targeting Rin4b. IGV screenshot of WGS reads mapping directly to the transgene sequence. WPT536-2 shows reads mapping to the transgene cassette, indicating a transgene insertion event. WPT536-2-13-15 and WPT536-2-13-16 do not have reads that match the transgene. The large regions of coverage across all three vectors (reads on the left side for all three genotypes) indicate the presence of the native Glycine max Ubiquitin promoter in the construct, which matches the Ubiquitin promoters (located at two separate loci) in the host genome.

## Slide 5
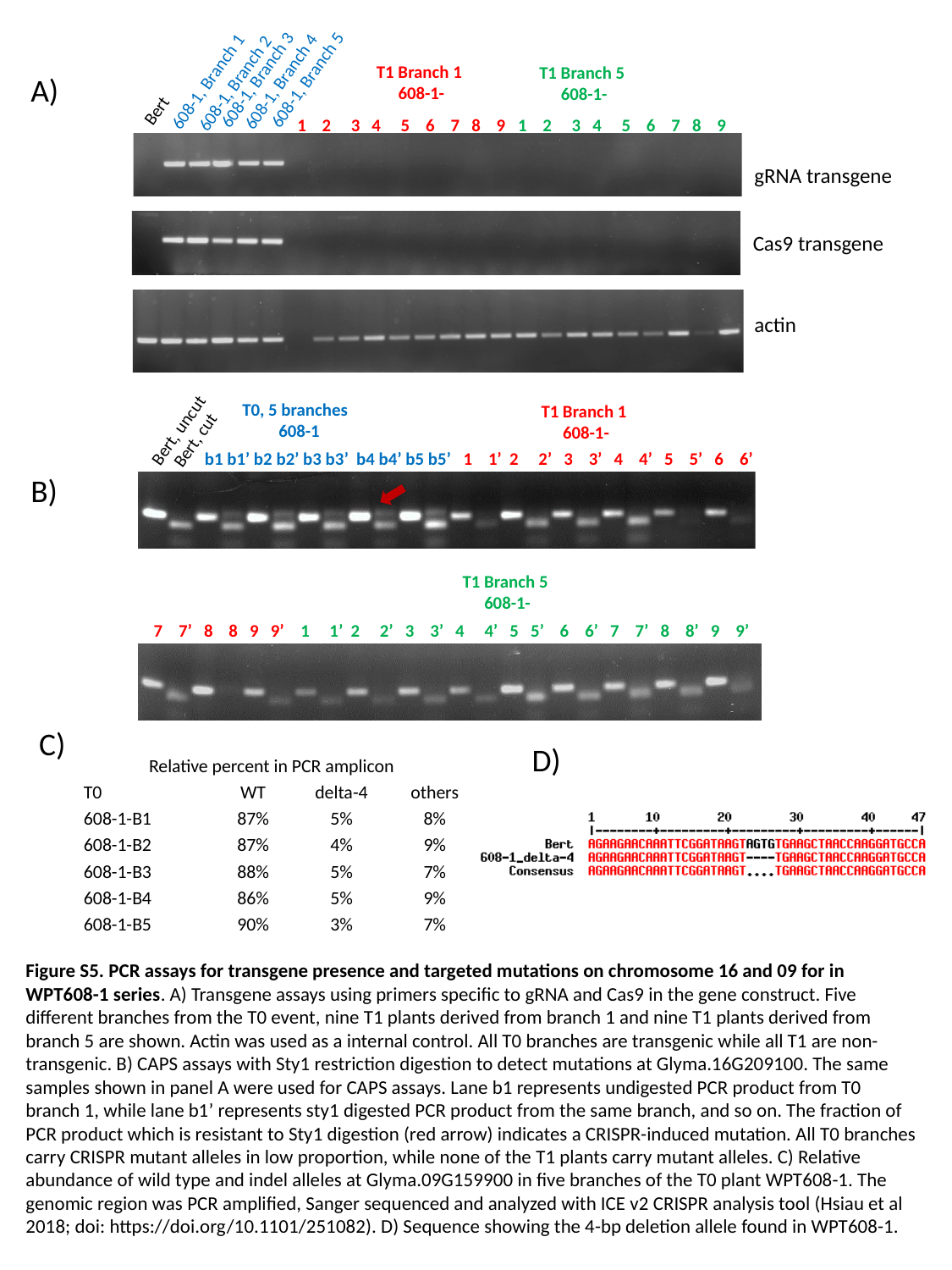

T1 Branch 1
608-1-
T1 Branch 5
608-1-
608-1, Branch 3
608-1, Branch 5
608-1, Branch 1
608-1, Branch 4
608-1, Branch 2
Bert
 1 2 3 4 5 6 7 8 9
 1 2 3 4 5 6 7 8 9
gRNA transgene
Cas9 transgene
actin
A)
T0, 5 branches
608-1
T1 Branch 1
608-1-
Bert, uncut
Bert, cut
 1 1’ 2 2’ 3 3’ 4 4’ 5 5’ 6 6’
 b1 b1’ b2 b2’ b3 b3’ b4 b4’ b5 b5’
T1 Branch 5
608-1-
 1 1’ 2 2’ 3 3’ 4 4’ 5 5’ 6 6’ 7 7’ 8 8’ 9 9’
 7 7’ 8 8 9 9’
B)
C)
D)
| Relative percent in PCR amplicon | | | | | |
| --- | --- | --- | --- | --- | --- |
| T0 | WT | delta-4 | others | | |
| 608-1-B1 | 87% | 5% | 8% | | |
| 608-1-B2 | 87% | 4% | 9% | | |
| 608-1-B3 | 88% | 5% | 7% | | |
| 608-1-B4 | 86% | 5% | 9% | | |
| 608-1-B5 | 90% | 3% | 7% | | |
Figure S5. PCR assays for transgene presence and targeted mutations on chromosome 16 and 09 for in WPT608-1 series. A) Transgene assays using primers specific to gRNA and Cas9 in the gene construct. Five different branches from the T0 event, nine T1 plants derived from branch 1 and nine T1 plants derived from branch 5 are shown. Actin was used as a internal control. All T0 branches are transgenic while all T1 are non-transgenic. B) CAPS assays with Sty1 restriction digestion to detect mutations at Glyma.16G209100. The same samples shown in panel A were used for CAPS assays. Lane b1 represents undigested PCR product from T0 branch 1, while lane b1’ represents sty1 digested PCR product from the same branch, and so on. The fraction of PCR product which is resistant to Sty1 digestion (red arrow) indicates a CRISPR-induced mutation. All T0 branches carry CRISPR mutant alleles in low proportion, while none of the T1 plants carry mutant alleles. C) Relative abundance of wild type and indel alleles at Glyma.09G159900 in five branches of the T0 plant WPT608-1. The genomic region was PCR amplified, Sanger sequenced and analyzed with ICE v2 CRISPR analysis tool (Hsiau et al 2018; doi: https://doi.org/10.1101/251082). D) Sequence showing the 4-bp deletion allele found in WPT608-1.

## Slide 6
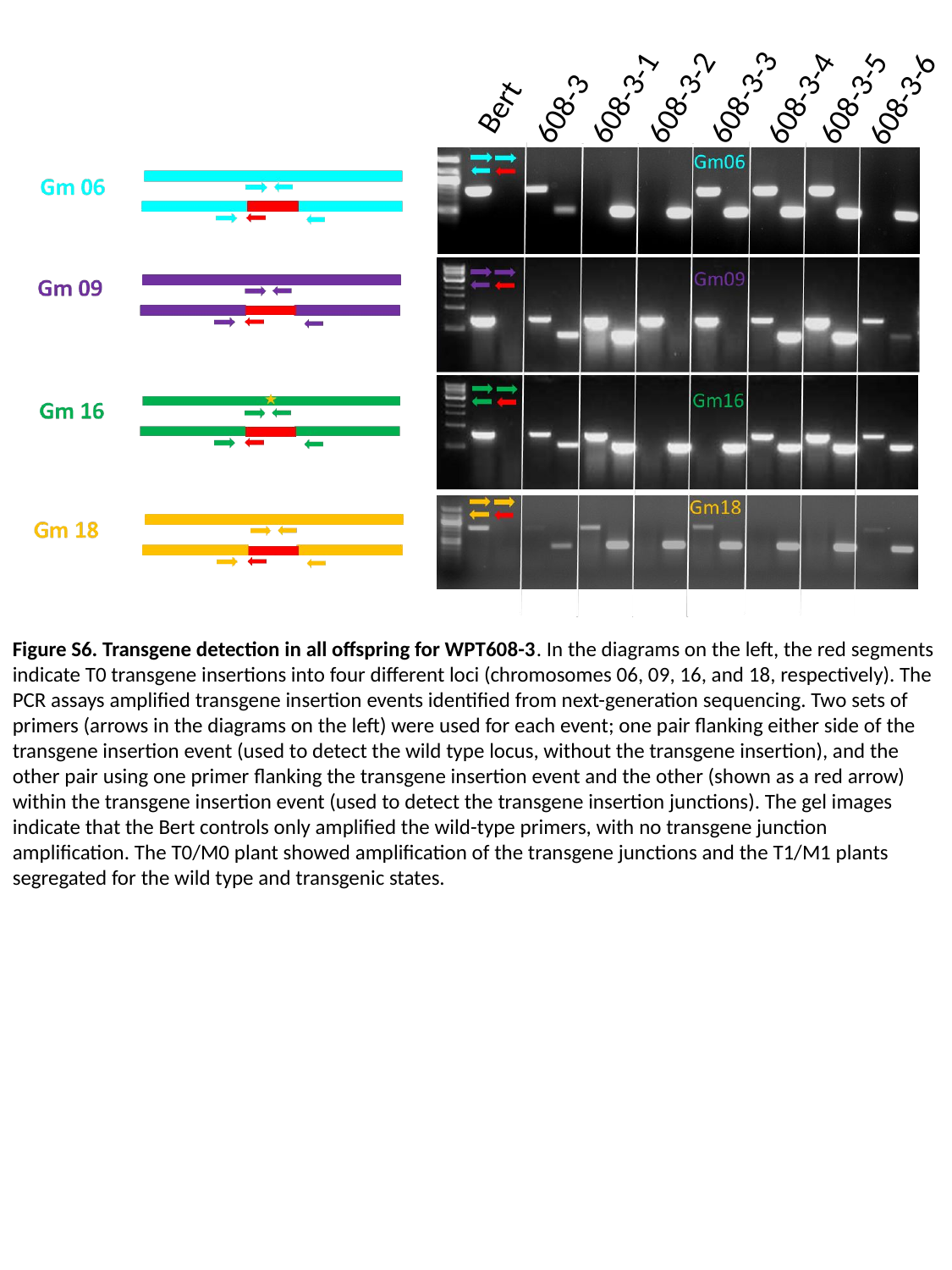

608-3-3
608-3-2
608-3-1
608-3-5
608-3-4
608-3-6
Bert
608-3
Figure S6. Transgene detection in all offspring for WPT608-3. In the diagrams on the left, the red segments indicate T0 transgene insertions into four different loci (chromosomes 06, 09, 16, and 18, respectively). The PCR assays amplified transgene insertion events identified from next-generation sequencing. Two sets of primers (arrows in the diagrams on the left) were used for each event; one pair flanking either side of the transgene insertion event (used to detect the wild type locus, without the transgene insertion), and the other pair using one primer flanking the transgene insertion event and the other (shown as a red arrow) within the transgene insertion event (used to detect the transgene insertion junctions). The gel images indicate that the Bert controls only amplified the wild-type primers, with no transgene junction amplification. The T0/M0 plant showed amplification of the transgene junctions and the T1/M1 plants segregated for the wild type and transgenic states.

## Slide 7
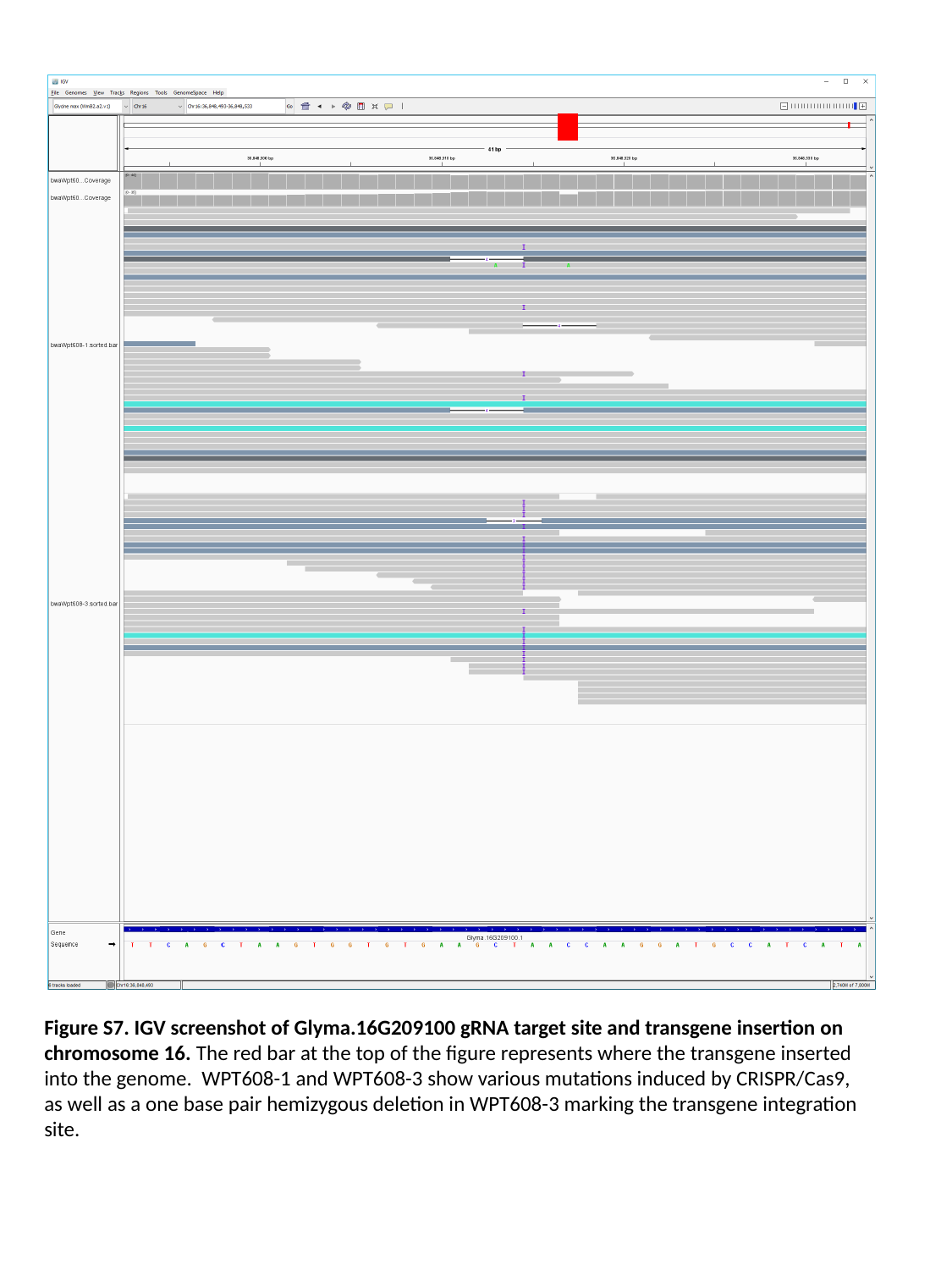

Figure S7. IGV screenshot of Glyma.16G209100 gRNA target site and transgene insertion on chromosome 16. The red bar at the top of the figure represents where the transgene inserted into the genome. WPT608-1 and WPT608-3 show various mutations induced by CRISPR/Cas9, as well as a one base pair hemizygous deletion in WPT608-3 marking the transgene integration site.

## Slide 8
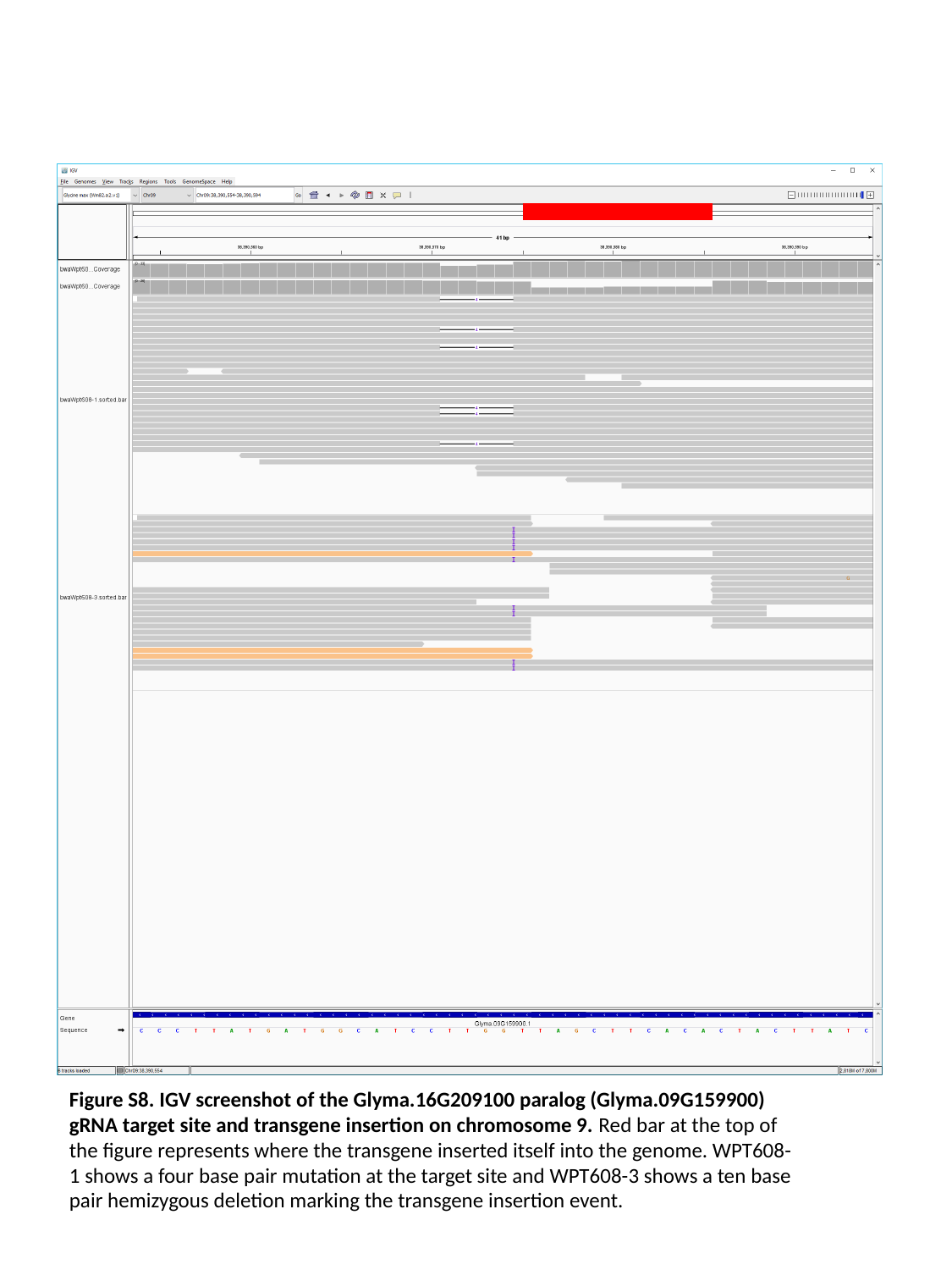

Figure S8. IGV screenshot of the Glyma.16G209100 paralog (Glyma.09G159900) gRNA target site and transgene insertion on chromosome 9. Red bar at the top of the figure represents where the transgene inserted itself into the genome. WPT608-1 shows a four base pair mutation at the target site and WPT608-3 shows a ten base pair hemizygous deletion marking the transgene insertion event.

## Slide 9
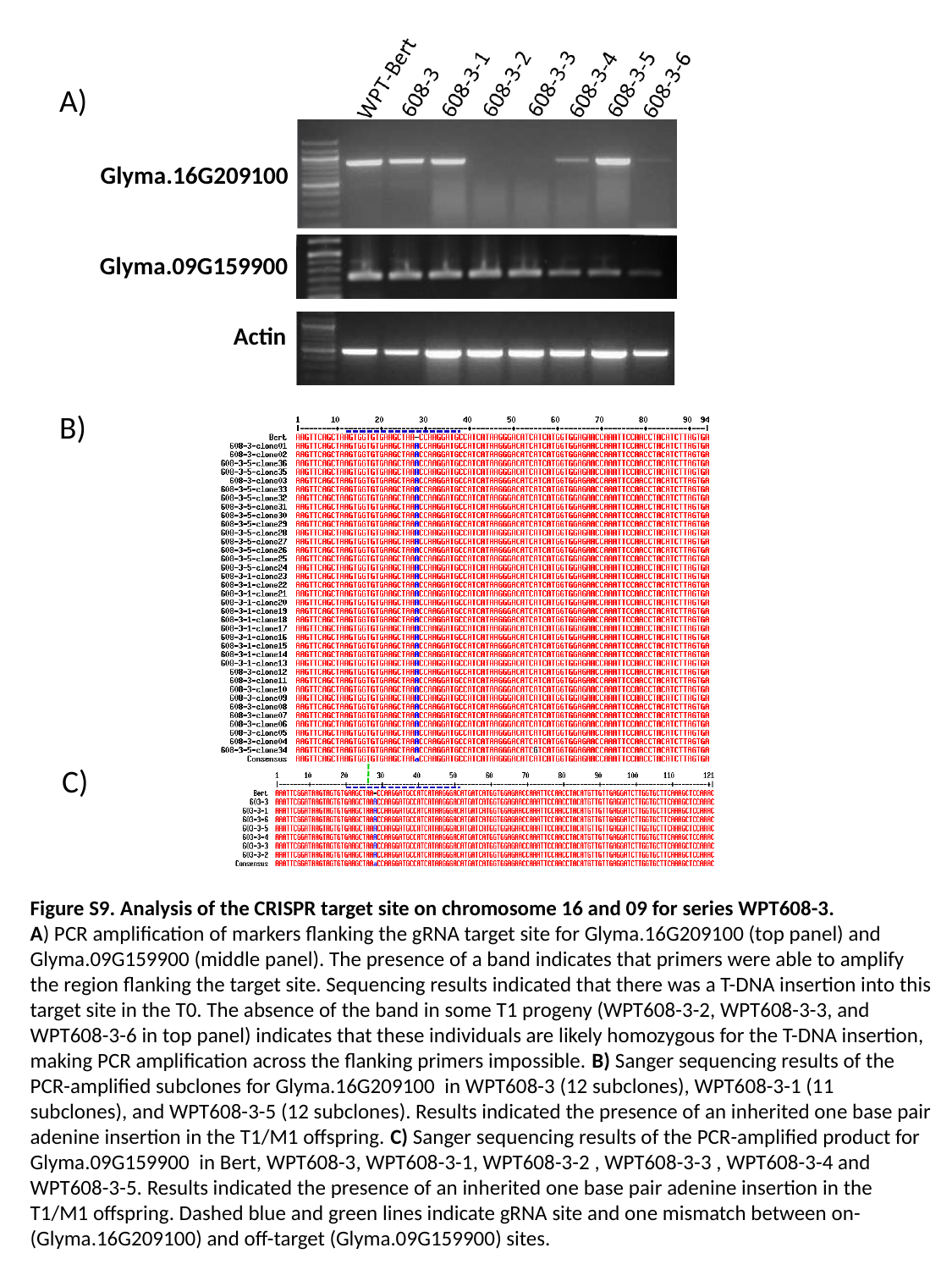

A)
Glyma.16G209100
Glyma.09G159900
Actin
B)
C)
Figure S9. Analysis of the CRISPR target site on chromosome 16 and 09 for series WPT608-3.
A) PCR amplification of markers flanking the gRNA target site for Glyma.16G209100 (top panel) and Glyma.09G159900 (middle panel). The presence of a band indicates that primers were able to amplify the region flanking the target site. Sequencing results indicated that there was a T-DNA insertion into this target site in the T0. The absence of the band in some T1 progeny (WPT608-3-2, WPT608-3-3, and WPT608-3-6 in top panel) indicates that these individuals are likely homozygous for the T-DNA insertion, making PCR amplification across the flanking primers impossible. B) Sanger sequencing results of the PCR-amplified subclones for Glyma.16G209100 in WPT608-3 (12 subclones), WPT608-3-1 (11 subclones), and WPT608-3-5 (12 subclones). Results indicated the presence of an inherited one base pair adenine insertion in the T1/M1 offspring. C) Sanger sequencing results of the PCR-amplified product for Glyma.09G159900 in Bert, WPT608-3, WPT608-3-1, WPT608-3-2 , WPT608-3-3 , WPT608-3-4 and WPT608-3-5. Results indicated the presence of an inherited one base pair adenine insertion in the T1/M1 offspring. Dashed blue and green lines indicate gRNA site and one mismatch between on- (Glyma.16G209100) and off-target (Glyma.09G159900) sites.

## Slide 10
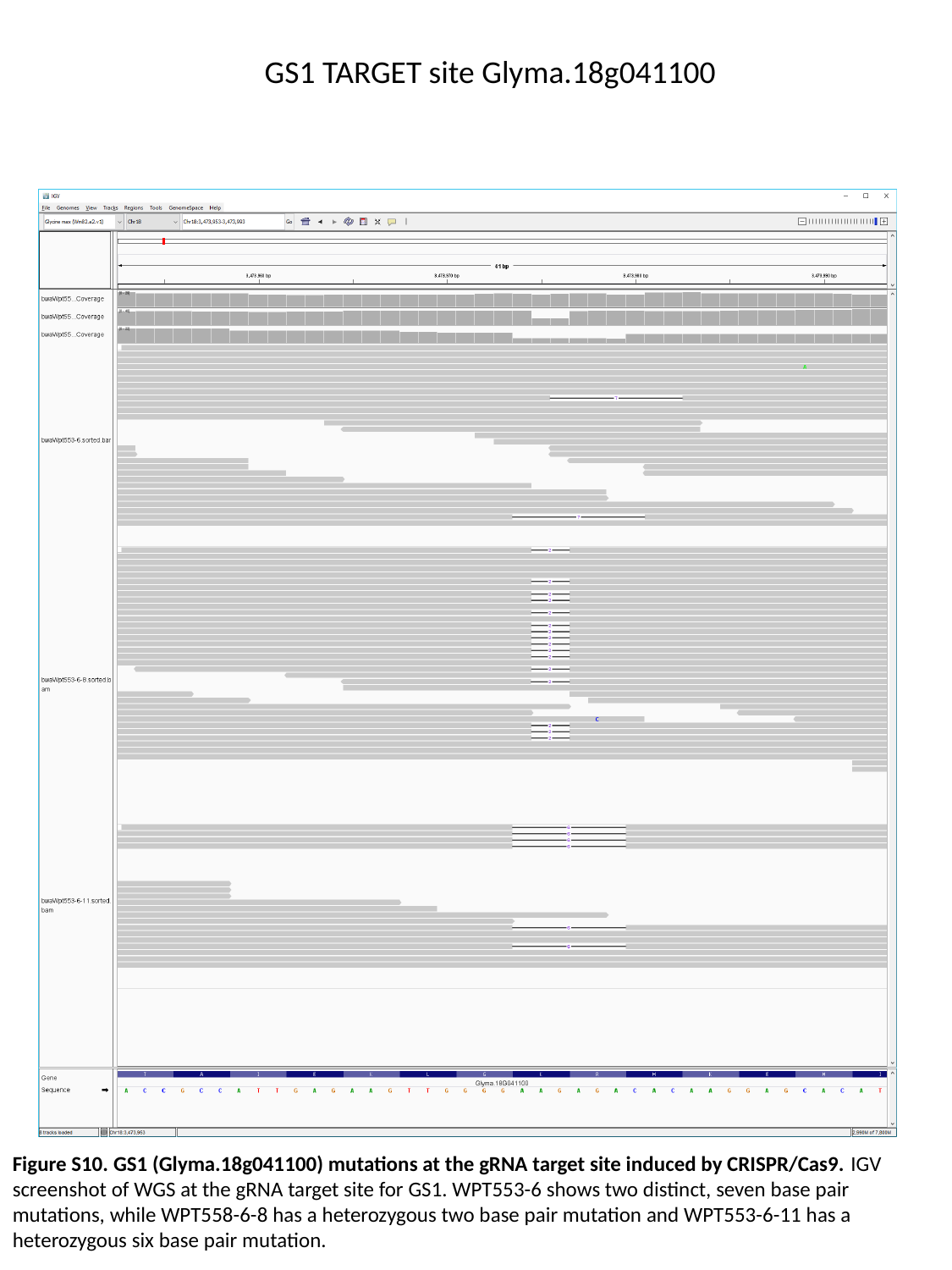

GS1 TARGET site Glyma.18g041100
Figure S10. GS1 (Glyma.18g041100) mutations at the gRNA target site induced by CRISPR/Cas9. IGV screenshot of WGS at the gRNA target site for GS1. WPT553-6 shows two distinct, seven base pair mutations, while WPT558-6-8 has a heterozygous two base pair mutation and WPT553-6-11 has a heterozygous six base pair mutation.

## Slide 11
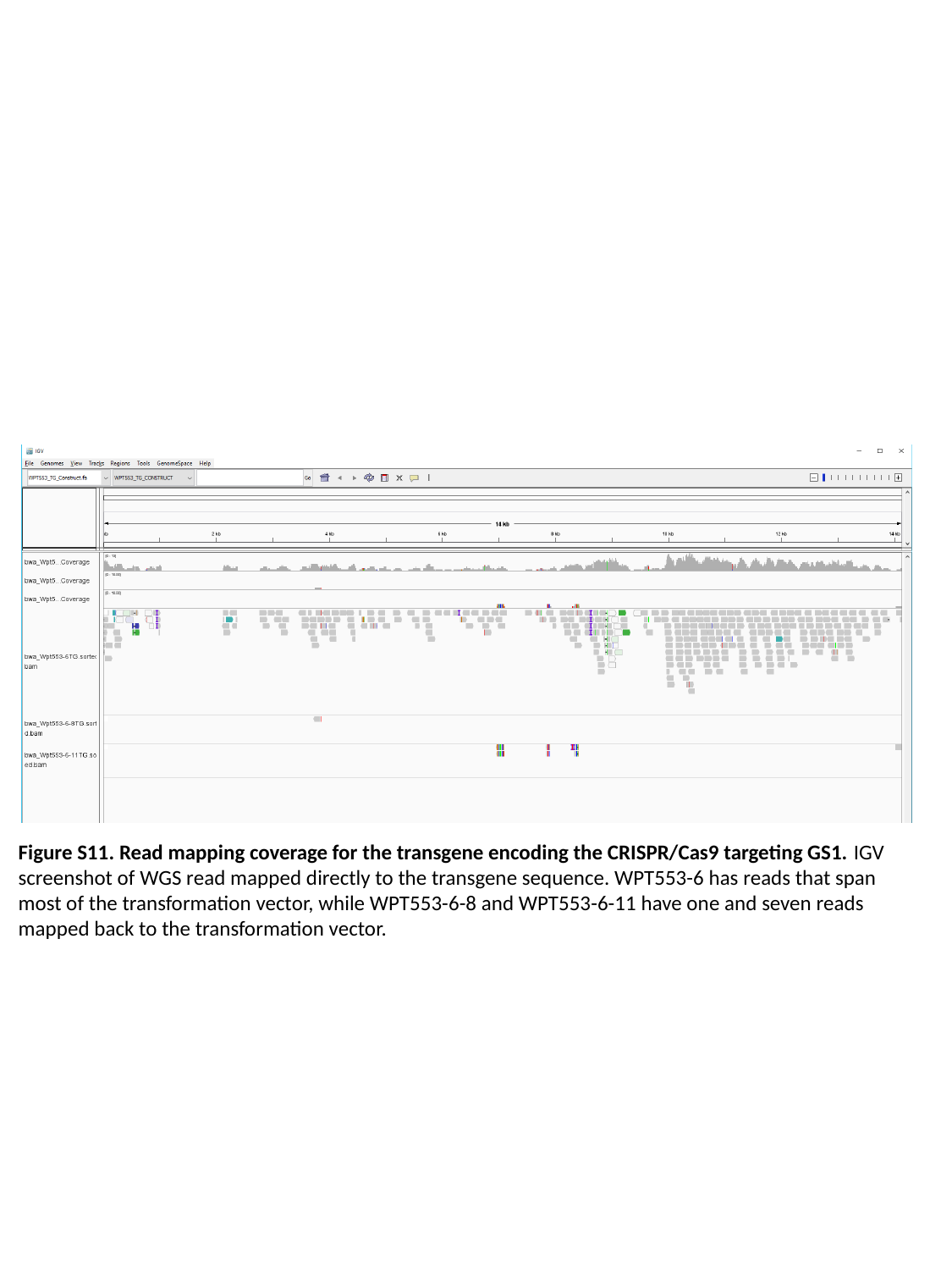

Figure S11. Read mapping coverage for the transgene encoding the CRISPR/Cas9 targeting GS1. IGV screenshot of WGS read mapped directly to the transgene sequence. WPT553-6 has reads that span most of the transformation vector, while WPT553-6-8 and WPT553-6-11 have one and seven reads mapped back to the transformation vector.
